# Development of a Multiplex Real-Time PCR Assay for the Simultaneous Detection of Two Fungal Pathogens Causing Pneumonia

**DOI:** 10.3390/jof10090619

**Published:** 2024-08-29

**Authors:** Ho-Jae Lim, Seojin Ahn, Jee-Hyun No, Min-Young Park, Min-Jin Kim, Yong-Hak Sohn, Kwang-Soo Shin, Jung-Eun Park, Yong-Jin Yang

**Affiliations:** 1 Department of Molecular Diagnostics, Seegene Medical Foundation, Seoul 04805, Republic of Korea; 52rotc.hjl@mf.seegene.com (H.-J.L.); seojin.ahn@mf.seegene.com (S.A.); jh_no@mf.seegene.com (J.-H.N.); pyli186@mf.seegene.com (M.-Y.P.); lithium2864@mf.seegene.com (M.-J.K.); medsohn@mf.seegene.com (Y.-H.S.); 2 Department of Microbiology, Graduate School, Daejeon University, Daejeon 34520, Republic of Korea; shinks@dju.kr; 3 Department of Integrative Biological Sciences & BK21 FOUR Educational Research Group for Age-Associated Disorder Control Technology, Chosun University, Gwangju 61452, Republic of Korea

**Keywords:** multiplex real-time PCR, molecular diagnostics, fungal respiratory infection, *Aspergillus fumigatus*, *Pneumocystis jirovecii*, diagnostic accuracy study

## Abstract

Infectious diseases caused by fungal sources are of great interest owing to their increasing prevalence. Invasive fungal infections, including invasive pulmonary aspergillosis caused by *Aspergillus fumigatus*, and *Pneumocystis* pneumonia caused by *Pneumocystis jirovecii*, are significant causes of morbidity and mortality among immunocompromised patients. The accurate and timely detection of these pathogens in this high-risk population is crucial for effective patient management. We developed a multiplex real-time polymerase chain reaction (PCR) assay, RF2 mRT-PCR, specifically designed to detect two respiratory fungi, *P. jirovecii* and *A. fumigatus*, and evaluated its performance in specimens of patients with lower respiratory tract infection. The performance was evaluated using 731 clinical samples, 55 reference species, and one synthetic DNA. The reproducibility test yielded a probit curve with a lower limit of detection of 19.82 copies/reaction for *P. jirovecii* and 64.20 copies/reaction for *A. fumigatus*. The RF2 mRT-PCR assay did not cross-react with non-*A. fumigatus Aspergillus* species or other common bacterial and viral species, and showed 100% in vitro sensitivity and specificity with reference assays. Additionally, it simultaneously detected *A. fumigatus* and *P. jirovecii* in co-infected samples. Therefore, the RF2 mRT-PCR assay is an efficient and reliable tool for in vitro diagnosis of *A. fumigatus* and *P. jirovecii* pulmonary infections.

## 1. Introduction

The World Health Organization (WHO) has reported that although prevention and preparedness measures for bacterial infections are well established, strategies to combat fungal infections are currently insufficient [1,2]. In December 2022, the WHO released a list of priority fungal pathogens threatening global health to bolster efforts in managing fungal infections. This list highlights the importance of *Aspergillus fumigatus*, which causes severe invasive fungal infections (IFIs), and *Pneumocystis jirovecii*, an opportunistic pathogen that causes life-threatening *Pneumocystis jirovecii* pneumonia (PJP) [2]. Infections caused by *A. fumigatus*, leading to invasive pulmonary aspergillosis (IPA), are the most common fungal pulmonary infection, with a mortality rate of 18.5% and over 1.8 million cases reported annually. PJP has a higher mortality rate of 42.4%, with 0.5 million cases reported annually [3].

*Aspergillus* spores typically enter the lower respiratory tract via inhalation, and infections sometimes originate in other areas such as the sinuses, the gastrointestinal tract, or the skin [4]. Similarly, *P. jirovecii* is transmitted via inhalation of airborne haploid spores [5], which are the main sources of fungal pulmonary infections in humans and can directly invade, causing fungal pneumonia [6]. Symptoms of fungal pneumonia, including cough, chills, fatigue, chest congestion, and sore throat, are generally indistinguishable from the symptoms of viral and bacterial pneumonia [7,8]. Nevertheless, clinicians rarely test for fungal infections because the incidence of fungal pneumonia is lower than that of viral and bacterial pneumonia [7].

Aspergillosis, the most frequent cause of fungal pulmonary infections, is caused predominantly by *A*. *fumigatus*, but also by other species such as *A. flavus*, *A. terreus*, and *A. niger* [9,10]. IPA, *A. fumigatus* is the pathogen in up to 89% of cases, and can be identified using microscopy, fungal culture, and blood tests [11]. PJP is diagnosed using lung tissue microscopy, polymerase chain reaction (PCR) tests, and blood tests. However, given the extreme difficulty in culturing *P. jirovecii* using standard methods [5], diagnosing both *P. jirovecii* and *A. fumigatus* infections typically requires specimens from patients with lower respiratory tract infections (LRTIs), such as bronchoalveolar lavage fluid (BALF), sputum, or lung tissue samples [4,12].

β-D-glucan (BDG) and PCR methods are used to diagnose both *P. jirovecii* and *A. fumigatus* infections [12,13,14]. However, BDG is nonspecific and detects a wide range of fungal antigens; therefore, more specific diagnostic tests are needed. Although these fungi are opportunistic pathogens and generally do not cause diseases in healthy individuals, they can lead to severe illness in immunosuppressed or immunocompromised individuals [15]. Few reports have been published on cases of *P. jirovecii* and *A. fumigatus* co-infection, but the available evidence suggests that co-infections are associated with higher mortality rates [15,16].

Furthermore, the diagnosis of cases of *P. jirovecii* and *A. fumigatus* co-infection requires separate detection of each pathogen using microscopy, galactomannan (GM), and PCR testing [17,18,19,20]. Therefore, a test that can detect both pathogens simultaneously is urgently needed. Multiplex real-time PCR (mRT-PCR) testing is a sensitive diagnostic tool that can detect multiple pathogens simultaneously, providing accurate results within a few hours and facilitating early diagnosis.

Previously, separate PCR-based assays have been used to detect *P. jirovecii*, *A. fumigatus*, and *Aspergillus* spp. [21,22,23,24,25]. However, conducting individual assays for multiple pathogens is time-consuming and resource-intensive, limiting the ability to simultaneously and rapidly detect these two major pathogens. Hence, this study aimed to develop an mRT-PCR assay for the rapid and reliable detection of two respiratory fungi (RF2), *P. jirovecii* and *A. fumigatus*. The diagnostic accuracy of the RF2 mRT-PCR assay was validated using LRTI specimens and by comparing the results to those from Sanger sequencing for detecting *A. fumigatus* and a commercial kit for detecting *P. jirovecii*.

## 2. Materials and Methods

### 2.1. Primer/Probe Design

The target genes of *P. jirovecii* and *A. fumigatus* were selected for primer and probe design based on the results of previous studies [26,27,28,29,30]. The analysis targeted the gene encoding the mitochondrial large subunit rRNA (*mtLSU*) for *P. jirovecii* and the 28S rRNA gene for *A. fumigatus*, with GenBank accession numbers NC_020331.1 and NG_055745.1, respectively, for the assigned DNA sequences. The species, including mutations and deletions, were accurately identified from large sequence databases of the National Center for Biotechnology Information (NCBI) using a conserved region. After identifying the conserved region, DNA oligomers were designed for either a dual-priming oligonucleotide (DPO)-based or TaqMan probe-based mRT-PCR assay. These designs were based on their stability and the ability to detect targets simultaneously, as determined in previous studies [31,32]. The melting temperature of the oligomers was calculated using GeneRunner ver 6.0 and in silico selectivity was verified using the NCBI Basic Local Alignment Search Tool (BLAST).

Phylogenetically closely related *Aspergillus* species in the section *Fumigati* were present [33]. Selectivity might be an issue for closely related *Aspergillus* species in the section *Fumigati*, including *A. lentulus*, *A. viridinutans*, *A. felis*, *A. udagawae*, *A. fumigatiaffinis*, *A. pseudofischeri*, *A. hiratsukae*, and *A. fischeri*, which had 1–2 bp mismatches for the oligonucleotides. Specificity was tested to differentiate *A. fumigatus* from other major pathogenic *Aspergillus* species, such as *A. nidulans*, *A. niger*, *A. terreus*, and *A. flavus* [34]. The primers used were as follows: forward for *P. jirovecii*: 5′-CTA GGA TAT AGC TGG TTT TCT GCG III IIT GTT TTG GCA-3′; reverse for *P. jirovecii*: 5′-AGC TTT AAT TAC TGT TCT GGG CTG III IIC TTT CGA CTA-3′; forward for *A. fumigatus*: 5′-GGG GTT CAG CCG GCA TTI III ICG GTG TAC TT-3′; reverse for *A. fumigatus*: 5′-GTT CCT CGG TCC AGG CAG GII III TTG CAC CCT C-3′; forward for the internal control: 5′-GGC ATA AAA GTC AGG GCA GAI III ICT ATT GCT-3′; reverse for the internal control: 5′-CCA ACT TCA TCC ACG TTC ACC III IIC CAC AGG G-3′); probes (*P. jirovecii*: 5′-TAG GTA TAG CAC TGA ATA TCT CGA GGG A-3′; *A. fumigatus*: 5′-CCT CGG AAT GTA TCA CCT CTC GG-3′; and the internal control: 5′-CCT GAG GAG AAG TCT GCC GTT ACT GC-3′. Further information on these primers is listed in Table 1.

### 2.2. Storage of Clinical Specimens

This study was approved by the Institutional Review Board of the Seegene Medical Foundation (SMF-IRB-2023-017). Residual lower respiratory specimens from 2332 patients, consisting of 1800 sputum and 532 BALF samples, were collected and preserved from March 2023 to June 2024 as part of routine diagnostic testing for *P. jirovecii* at the Seegene Medical Foundation (Seoul, Republic of Korea).

### 2.3. Nucleic Acid Extraction

Sputum samples were homogenized in 1–3 mL of 1X phosphate-buffered saline (pH 7.2), as described previously [35]. After thawing, BALF specimens were used directly without preprocessing. A 200 µL aliquot of sputum or BALF was transferred to the processing cartridge. Nucleic acids from the 2332 samples were isolated using the MagNA Pure 96 system (Roche, Basel, Switzerland) for subsequent PCR, as described previously [36].

### 2.4. Aspergillus spp. Screening and Aspergillus fumigatus—Specific PCR and Sanger Sequencing

The 18S rRNA genes of the *Aspergillus* species were amplified using primers modified from a previous study [37]. These primers (forward: 5′-CCA GCG AGT ACA TCA CCT TGG G-3′; reverse: 5′-TCC RTT GTT GAA AGT TTT IAC TGA TT-3′) bind to conserved regions of the fungal 18S small-subunit rRNA (Supplementary Table S1). Additionally, the 5.8S rRNA gene of *A. fumigatus* was specifically amplified using primers modified from a previous study that bind exclusively to *A. fumigatus* (forward: 5′-GGC CCG CCG TTT CGA C-3′ and reverse: 5′-GCC CCA TAC GCT CGA GGA-3′) [38]. Both PCR assays were conducted under identical conditions: 10 pmol of forward and reverse primers, an initial denaturation step (94 °C, 15 min), followed by 40 cycles of denaturation (94 °C, 30 s), annealing (60 °C, 1 min), and extension (72 °C, 1 min), with a final extension step (72 °C, 10 min). The amplicons were purified, dye-labeled, and analyzed. Fasta sequences were analyzed using NCBI-BLAST, and those with over 95% accuracy were used as a reference [39].

### 2.5. RF2 mRT-PCR and Control Isolates

RF2 mRT-PCR testing was performed using a CFX96 instrument (Bio-Rad Laboratories, Hercules, CA, USA) with a reaction volume of 20 μL, consisting of 10 μL of an oligonucleotide mixture (forward primer 10 pmol, reverse primer 10 pmol, *P. jirovecii* probe 3 pmol, *A. fumigatus* probe 5 pmol, and internal control probe 10 pmol), 5 μL of TOPreal Multi-Probe qPCR PreMIX (Enzynomics, Daejeon, Republic of Korea), and 5 μL of nucleic acid. The RF2 mRT-PCR assay was run with the following parameters: a first step (50 °C, 20 min), an initial denaturation step (95 °C, 15 min), followed by 45 cycles of denaturation (95 °C, 10 s), annealing (60 °C, 1 min), and final extension (72 °C, 10 s). The test runs were validated based on the positive control for each amplification and the absence of amplification in the negative control (no template). The RF2 mRT-PCR assay was designed to detect *P. jirovecii* in the CalRed 610 channel and *A. fumigatus* in the FAM channel. To prevent false-negative results, human hemoglobin subunit beta (*HBB*) was concurrently amplified as an endogenous internal control, as described previously [35]. The *P. jirovecii*-positive laboratory samples and Zeptometrix-Z014 *A. fumigatus* were amplified using RF2 mRT-PCR primers. Subsequently, the amplified DNA underwent cloning to generate positive controls as described previously [35]. This process involved ligation and TA cloning, followed by heat shocking the constructed plasmid into competent DH5α cells.

### 2.6. Analytical Performance

To test the analytical specificity of the RF2 mRT-PCR assay, 55 species of fungi, bacteria, and viruses were used. These included respiratory pathogens, comprising 8 strains of fungi, 12 strains of bacteria, and 19 strains of viruses; non-respiratory pathogens, including 12 strains of bacteria and 4 strains of viruses; and one synthetic DNA sequence of *P. jirovecii*. The strains used in this study were obtained from Zeptometrix (Buffalo, NY, USA), the American Type Culture Collection (ATCC; Manassas, VA, USA), the Korean Collection for Type Cultures (KCTC; Jeongeup, Republic of Korea), the Korea National Research Resource Center (KNRRC; Seoul, Republic of Korea), the National Institute for Biological Standards and Control (NIBSC; Potters Bar, UK), the Korean Culture Center of Microorganisms (KCCM; Seoul, Republic of Korea), the National Culture Collection for Pathogens (NCCP; Cheongju, Republic of Korea), and the Korea Bank for pathogenic viruses (KBPV; Seoul, Republic of Korea).

The analytical sensitivity for *P. jirovecii* and *A. fumigatus* was determined by testing the serial dilution of the positive controls. Each control sample was serially diluted to 10^4^, 10^3^, 10^2^, 10^1^, and 1 copy/reaction, and 40 replicates were performed.

### 2.7. Clinical Performance of RF2 mRT-PCR Assay

Of the archived samples, 2332 were either positive (*n* = 331) or negative (*n* = 2001) for *P. jirovecii*. The collected samples were stored at −80 °C without homogenization until further testing. Positive samples obtained from these 2332 samples were subsequently screened for *Aspergillus*.

Approximately 31% (731/2332) of the total samples screened for *Aspergillus* spp. were evaluated for clinical performance using a commercial kit for *P. jirovecii* and Sanger sequencing for *A. fumigatus*-specific primers, respectively. The clinical performance was verified using *P. jirovecii*-positive samples and 20% of *P. jirovecii*-negative samples (the convenience sampling method). The diagnostic performance of the RF2 mRT-PCR assay was assessed using a RealStar *P. jirovecii* PCR kit 1.0 (altona Diagnostics GmBH, Hamburg, Germany) as the reference test for *P. jirovecii* and Sanger sequencing specifically for *A. fumigatus* as the reference test for *A. fumigatus*.

The RealStar *P. jirovecii* PCR kit 1.0, validated on sputum, BALF, and lung tissue specimens, targets the *mtLSU* and allows for the specific detection of *P. jirovecii* DNA. A CFX96 instrument was used to perform PCR testing on the clinical samples according to the manufacturer’s instructions.

### 2.8. Statistical Analysis

Data analyses were performed using R version 4.2.2 (the R Foundation for Statistical Computing, Vienna, Austria) for Windows. The statistical analysis in this study included calculating the lower limit of detection (LOD) for *P. jirovecii* and *A. fumigatus* using probit analysis corresponding to a probability of 0.95 [40]. The in vitro sensitivity and specificity were determined by comparing the RF2 mRT-PCR results to those of the reference assays. Cohen’s kappa values were used to measure the agreement between the RF2 mRT-PCR assay and the reference tests using the DescTools package ver. 0.99.56 [41].

## 3. Results

### 3.1. Analytical Specificity of the RF2 mRT-PCR Assay

The specificity of the RF2 mRT-PCR assay was confirmed using 55 species and one synthetic DNA. No cross-reactivity was observed between *A. fumigatus* and five non-*A. fumigatus Aspergillus* species, namely *A. flavus*, *A. niger*, *A. terreus*, *A. nidulans*, and *A. versicolor* (Table 2). Additionally, the RF2 mRT-PCR assay exhibited 100% specificity for detecting *A. fumigatus* and *P. jirovecii* when used to test 33 other respiratory pathogens (*Penicillium chrysogenum* and *Talaromyces marneffei for fungi*, 12 bacteria, and 19 viruses; Table 2), and 16 non-respiratory pathogens (12 bacteria and four viruses; Supplementary Table S2).

### 3.2. The Analytical Sensitivity and LOD of the RF2 mRT-PCR Assay

**Figure 1:**
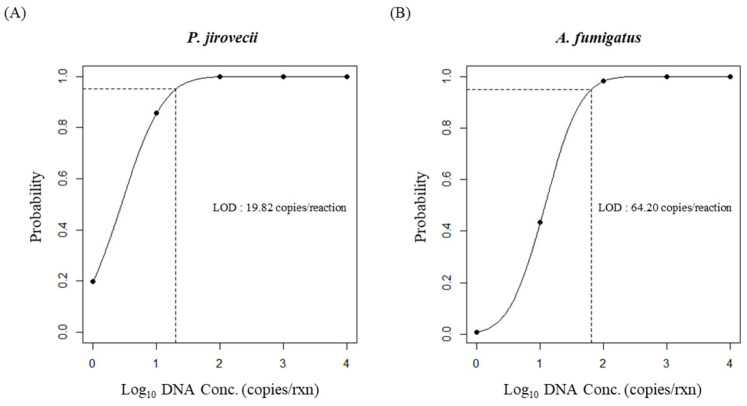
The limit of detection (LOD) of the RF2 mRT-PCR assay. The LOD of the RF2 mRT-PCR for both (**A**) *Pneumocystis jirovecii* and (**B**) *Aspergillus fumigatus* was calculated using a probit curve. A ten-fold dilution series of synthetic DNA ranging from 1 to 10^4^ copies/reaction was tested for 40 replicates each. The LOD at 95% was extrapolated from the sigmoid curve.

Serial dilutions of synthetic DNA, ranging from 1 to 10^4^ copies/reaction, were prepared to determine the lower LOD for *P. jirovecii* and *A. fumigatus* (Figure 1). The analytical sensitivity was observed to be 100% reproducible for concentrations ranging from 10^2^ to 10^4^ copies/reaction, with variable results for dilution at or below 10^1^ copies/reaction: *P. jirovecii* for 10^1^, 85% (34/40); *P. jirovecii* for 1, 20% (8/40); *A. fumigatus* for 10^1^, 37.5% (15/40); and *A. fumigatus* for 1, 2.5% (1/40). The 95% LOD obtained from the probability plots was 19.82 copies/reaction for *P. jirovecii* and 64.20 copies/reaction for *A. fumigatus*. The RF2 mRT-PCR assay exhibited a 95% LOD ranging from 10^1^ to 10^2^ copies/reaction.

### 3.3. Aspergillus and P. jirovecii Co-Infection Rates in Clinical Samples

**Figure 2:**
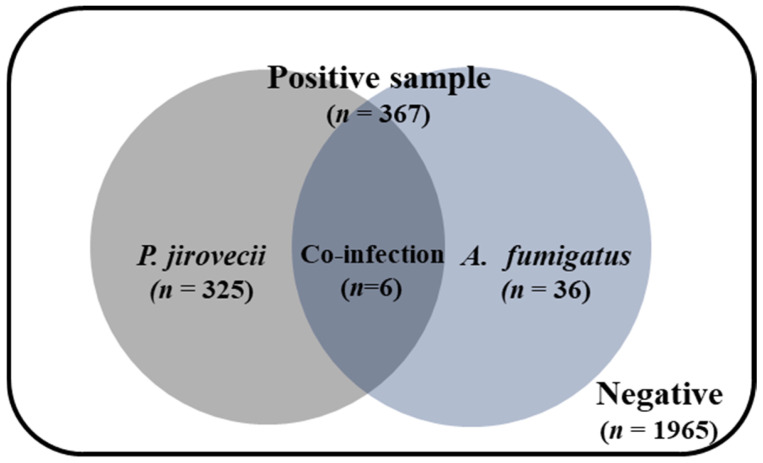
The distribution of *A. fumigatus* detected using *Aspergillus* species sequencing with *P. jirovecii* results. A total of 2332 samples were tested using *Aspergillus* spp. primers with Sanger sequencing.

In this study, 2332 samples were collected, among which 64 (2.7%) tested positive for *Aspergillus* (Supplementary Table S3). Of the 64 samples positive for *Aspergillus* spp., 42 were identified as *A. fumigatus*, 11 as *A. nidulans*, 8 as *A. niger*, 3 as *A. flavus,* and 1 as *A. terreus*, with one sample being positive for both *A. fumigatus* and *A. terreus*. *A. fumigatus* accounted for 65% (42/65) of the *Aspergillus*-positive cases. Among the 42 *A. fumigatus*-positive samples, 6 samples were also positive for *P. jirovecii* (Figure 2). These findings suggest that some patients from whom the samples were collected were co-infected with *A. fumigatus* and *P. jirovecii*.

### 3.4. Comparison of RF2 mRT-PCR with the Reference Assays Using Clinical Specimens

Among the 731 clinical specimens tested using the RF2 mRT-PCR assay, 331 samples were positive for *P. jirovecii* and 42 samples for *A. fumigatus*, including 6 samples positive for both *P. jirovecii* and *A. fumigatus*. Regarding the reference assays, 331 samples were positive for *P. jirovecii* using the RealStar *P. jirovecii* PCR kit 1.0, and 42 samples were positive for *A. fumigatus* using the *A. fumigatus*-specific Sanger sequencing assay, whereas 23 samples with the non-*A. fumigatus Aspergillus* species tested negative. The results of the RF2 mRT-PCR assay exhibited 100% concordance with those of the *P. jirovecii* and *A. fumigatus* reference assays in all the samples tested (Table 3 and Table 4). Thus, the in vitro sensitivity and specificity of the RF2 mRT-PCR assay were 100% for both *P. jirovecii* and *A. fumigatus*, indicating high diagnostic accuracy in clinical samples compared with other molecular methods.

## 4. Discussion

Respiratory infections with outbreak potential continue to pose a global threat to human health [42]. However, in contrast to epidemics caused by viral and bacterial pathogens, fungal infections represent a silent epidemic, primarily affecting immunocompromised individuals such as organ transplant recipients and patients undergoing immunotherapy [43]. Therefore, continuous surveillance, reliable diagnostic testing, and appropriate treatment are necessary for the effective prevention and control of fungal infections [44]. The development of the RF2 mRT-PCR assay marks a significant advancement in the detection of both *P. jirovecii* and *A. fumigatus* in LRTI specimens. Our findings indicate that the RF2 assay exhibits high analytical specificity for 55 species and one synthetic DNA, making it a reliable detection tool for both *A. fumigatus* and *P. jirovecii*.

The number of deaths due to IFIs caused by *Pneumocystis* and *Aspergillus* infections, which most frequently present as fungal pneumonia, is increasing [45,46]. In patients with fungal pneumonia, the nonspecific clinical manifestations and co-infections with various other pathogens hinder accurate diagnosis. Conventional methods, such as culture and microscopy, are time-consuming and have limited diagnostic accuracy [45].

No methods are available for *P. jirovecii* culture, and fungal culture for *A. fumigatus* takes 7 days [5,47]. Morphological hallmarks based on macroscopic and microscopic characteristics can be used to differentiate filamentous fungi, including *Aspergillus* species, from non-filamentous fungi [47]. Another approach to the diagnosis of fungal infections involves the serological detection of antibodies to a common fungal component, such as the BDG test [17]. Although serological methods generally exhibit good sensitivity and specificity, their ability to identify specific species is limited [48,49].

Currently, the GM method is a standard method for diagnosing IFIs. Unlike BDG, *Pneumocystis* spp. and *Aspergillus* spp. can be distinguished, although the turnaround time varies [50]. A gene-based molecular assay provides reliable diagnosis for *P. jirovecii* and *A. fumigatus*, providing test results within hours. In this study, the RF2 mRT-PCR assay ensured the completion of the reactions within 3 h, offering a rapid and accurate diagnostic tool.

Molecular assays include next-generation sequencing, Sanger sequencing, and PCR testing [51]. During the COVID-19 pandemic, PCR testing was used to accurately detect severe acute respiratory syndrome coronavirus 2 [52]. In this study, *Aspergillus* species were screened using Sanger sequencing of samples from 2332 patients with suspected PJP, of which 1.8% tested positive for *A. fumigatus* and 1.0% tested positive for other (non-*A. fumigatus*) *Aspergillus* species. However, the *Aspergillus* genus comprises various species, that primarily manifest as pneumonia clinically. Therefore, differentiation between *A. fumigatus* and other *Aspergillus* species in clinical samples is difficult. A previous study demonstrated that DPO increases specificity [31], highlighting the need for evaluation using clinical samples to accurately assess specificity. In the present study, the DPO-based RF2 mRT-PCR assay demonstrated 100% in vitro specificity using clinical samples, including 23 samples containing four non-*A. fumigatus Aspergillus* species.

Under optimal conditions, the primer pairs exhibit high performance in terms of analytical sensitivity and amplification efficiency [53]. The LOD of the RF2 mRT-PCR assay for the detection of *P. jirovecii* and *A. fumigatus* was 19.82, and 64.20 copies/reaction, respectively, comparable to the performance of alternative assays reported in previous studies [28,54]. Notably, compared with reference molecular assays, the in vitro sensitivity of the RF2 mRT-PCR assay was 100% using clinical samples.

This study has some limitations. First, to ensure accuracy in the diagnosis of IFIs, additional assays are needed to detect non-*A. fumigatus Aspergillus* species and other fungi, including *Cryptococcus neoformans* and *Cryptococcus gattii*. Second, although the performance of the RF2 mRT-PCR assay was validated using a commercial kit for *P. jirovecii*, *A. fumigatus* was validated using Sanger sequencing because no suitable commercial kit was available for evaluation. Third, other specimen types, such as serum and tissue samples, which can be used to detect *A. fumigatus* and *P. jirovecii* were not tested. Finally, the clinical manifestations and diagnostic accuracy of standard diagnostic methods were not evaluated. Therefore, more assays for fungal pathogens should be developed, and their performance should be compared with that of additional commercial kits for *A. fumigatus*, as well as standard diagnostic methods (GM, methenamine silver stain, or Giemsa stain) and according to patient clinical characteristics.

## 5. Conclusions

Owing to the increasing incidence of IFIs caused by *P. jirovecii* and *A. fumigatus* worldwide, the accurate and timely detection of the causative fungus is crucial for managing immunocompromised patients and improving outcomes through the use of appropriate treatment. In this study, we confirmed that the RF2 mRT-PCR assay was specific for *A. fumigatus* and that it did not cross-react with other *Aspergillus* species. Furthermore, the in vitro sensitivity and specificity of the RF2 mRT-PCR assay were 100%, enabling the simultaneous detection of co-infection with *P. jirovecii* and *A. fumigatus*. This study suggests that the RF2 mRT-PCR assay can be used as an in vitro diagnostic test to screen for fungal pneumonia caused by IFIs. Nevertheless, additional studies using comparative analyses of standard methods are required to determine the efficiency and added value of the assay.

## Figures and Tables

**Table 1:** Oligonucleotide primers and probes for real-time PCR detection of target pathogens and internal control.

Pathogen	Target Gene	Primer	Sequences (5′–3′)	Tm (°C)
*P. jirovecii*	*mtLSU-rRNA*	Fwd.	CTAGGATATAGCTGGTTTTCTGCGIIIIITGTTTTGGCA	60
		Rev.	AGCTTTAATTACTGTTCTGGGCTGIIIIICTTTCGACTA	59.5
		Probe	Cal Red 610-TAGGTATAGCACTGAATATCTCGAGGGA	64
*A. fumigatus*	*28S rRNA*	Fwd.	GGGGTTCAGCCGGCATTIIIIICGGTGTACTT	59.7
		Rev.	GTTCCTCGGTCCAGGCAGGIIIIITTGCACCCTC	61.2
		Probe	FAM-CCTCGGAATGTATCACCTCTCGG	64.2
Internal control	*HBB*	Fwd.	GGCATAAAAGTCAGGGCAGAIIIIICTATTGCT	56.9
		Rev.	CCAACTTCATCCACGTTCACCIIIIICCACAGGG	59.0
		Probe	HEX-CCTGAGGAGAAGTCTGCCGTTACTGC	68.8

Probes were labeled with FAM, HEX, and Cal Red 610 and detected at 518, 556, and 610 nm, respectively. All the probes had a Black Hole Quencher (BHQ) as a quencher at the 3′ end. Abbreviations: *mtLSU-rRNA*, mitochondrial large subunit ribosomal RNA; *28S rRNA*, 28S ribosomal RNA; *HBB*, hemoglobin subunit beta; Tm, melting temperature; probe, fluorescently labeled primer; and I, inosine.

**Table 2:** The specificity of the RF2 mRT-PCR assay for detecting *Pneumocystis jirovecii* and *Aspergillus fumigatus* when challenged using a range of respiratory pathogens.

Group	Organism	Source	Catalog No.	*P. jirovecii*	*A. fumigatus*
Fungi	*Pneumocystis jirovecii*	Synthetic DNA	-	positive	negative
	*Aspergillus fumigatus*	Zeptometrix	Z014	negative	positive
	*Aspergillus flavus*	Zeptometrix	Z013	negative	negative
	*Aspergillus niger*	Zeptometrix	Z105	negative	negative
	*Aspergillus terreus*	Zeptometrix	Z016	negative	negative
	*Aspergillus nidulans*	ATCC	38163	negative	negative
	*Aspergillus versicolor*	ATCC	11730	negative	negative
	*Penicillium chrysogenum*	KCCM	11609	negative	negative
	*Talaromyces marneffei*	KCCM	60287	negative	negative
Bacteria	*Streptococcus pneumoniae*	ATCC	49619	negative	negative
	*Legionella pneumophila*	KCTC	12009	negative	negative
	*Bordetella pertussis*	KCCM	42710	negative	negative
	*Bordetella parapertussis*	ATCC	15311	negative	negative
	*Mycoplasma pneumoniae*	ATCC	29342	negative	negative
	*Chlamydophila pneumoniae*	ATCC	53592	negative	negative
	*Haemophilus influenzae*	ATCC	9007	negative	negative
	*Pseudomonas aeruginosa*	KCCM	11266	negative	negative
	*Staphylococcus aureus*	KCCM	32395	negative	negative
	*Klebsiella pneumoniae*	KCCM	42750	negative	negative
	*Moraxella catarrhalis*	KCCM	42706	negative	negative
	*Streptococcus pyogenes*	ATCC	19615	negative	negative
Virus	Respiratory syncytial virus A	ATCC	VR-1803	negative	negative
	Respiratory syncytial virus B	ATCC	VR-955	negative	negative
	Influenza A virus	ATCC	VR-810	negative	negative
	Influenza B virus	Zeptometrix	0810255CF	negative	negative
	Parainfluenza type 1 virus	ATCC	VR-1380	negative	negative
	Parainfluenza type 2 virus	ATCC	VR-92	negative	negative
	Parainfluenza type 3 virus	ATCC	VR-93	negative	negative
	Parainfluenza type 4 virus	Zeptometrix	0810060CF	negative	negative
	Enterovirus A	KBPV	VR-10	negative	negative
	Adenovirus type 3	Zeptometrix	0810062CF	negative	negative
	Metapneumovirus 27	Zeptometrix	0810164CF	negative	negative
	SARS-CoV-2	NCCP	43330	negative	negative
	Coronavirus NL63	Zeptometrix	0810228CFHI	negative	negative
	Coronavirus 229E	ATCC	VR-740	negative	negative
	Coronavirus OC43	ATCC	VR-1558	negative	negative
	Rhinovirus	Zeptometrix	0810285CF	negative	negative
	Dengue virus type 2	ATCC	VR-1584	negative	negative
	Dengue virus type 4	ATCC	VR-1257CAF	negative	negative
	Echovirus 30	KNRRC	45	negative	negative

RF2 mRT-PCR assays were performed using 39 species and 1 synthetic DNA corresponding to the target species. Abbreviations: ATCC, American Type Culture Collection; KBPV, Korea Bank for pathogenic viruses; KCTC, Korean Collection for Type Cultures; KCCM, Korean Culture Center of Microorganisms; KNRRC, Korea National Research Resource Center; NCCP, National Culture Collection for Pathogens; and SARS-CoV-2, severe acute respiratory syndrome coronavirus 2.

**Table 3:** Comparison of the quantification of clinical *Pneumocystis jirovecii* using a commercial kit and the RF2 mRT-PCR assay.

Assay		RealStar *P. jirovecii* PCR Kit 1.0		Sensitivity (%)	Specificity (%)	κ	*p*-Value
		Positive	Negative				
RF2 mRT-PCR	Positive	331	0	100	100	1.0	<0.001
	Negative	0	400				

Analysis of the in vitro sensitivity, in vitro specificity, kappa value (κ), and *p*-value, assessed using the RF2 mRT-PCR assay versus the reference assay for *P. jirovecii*. Abbreviation: RF2 mRT-PCR, two respiratory fungi multiplex real-time polymerase chain reaction.

**Table 4:** Comparison of the quantification of clinical *Aspergillus fumigatus* using Sanger sequencing and the RF2 mRT-PCR assay.

Assay	Sanger Sequencing for *A. fumigatus*		
	Positive	Negative (*Aspergillus* spp.)	Negative (non-*Aspergillus* spp.)
**RF2 mRT-PCR**			
Positive (*n*)	42	0	0
Negative (*n*)	0	23	666
Sensitivity (%)	100		
Specificity (%)	100		
κ	1.0		
*p*-value	<0.001		

Analysis of the in vitro sensitivity, in vitro specificity, kappa value (κ), and *p*-value, assessed using the RF2 mRT-PCR assay versus the reference assay for *A. fumigatus*. Abbreviation: RF2 mRT-PCR, two respiratory fungi multiplex real-time polymerase chain reaction.

## Data Availability

The datasets used and/or analyzed during the current study are available from the corresponding author upon reasonable request.

## Supplementary Materials

The following supporting information can be downloaded at: https://www.mdpi.com/article/10.3390/jof10090619/s1, Supplementary Table S1: Oligonucleotide primers for differentiating *Aspergillus fumigatus* from other *Aspergillus* spp. pathogens; Supplementary Table S2: The specificity of the RF2 mRT-PCR assay for detecting *Pneumocystis jirovecii* and *Aspergillus fumigatus* when challenged using a range of non-respiratory pathogens; Supplementary Table S3: Distribution of *Aspergillus species* detected using Sanger sequencing.
