# Supplementary material for: Development of a Multiplex Real-Time PCR Assay for the Simultaneous Detection of Two Fungal Pathogens Causing Pneumonia
Source: J Fungi (Basel). 2024 Aug 29;10(9):619. doi: 10.3390/jof10090619 (PMC11433024; doi:10.3390/jof10090619)
Supplement: Supplementary file 1 [file jof-10-00619-s001.zip › jof-3122120-supplementary.pdf]

Journal of *Fungi*

MDPI

Supplementary Materials and Methods

Development of a multiplex real-time PCR assay for the simultaneous detection of two fungal pathogens causing pneumonia

Supplementary Table S1. Oligonucleotide primers for differentiating *Aspergillus fumigatus* from other *Aspergillus* spp. pathogens.

Pathogen	Target gene	Primer	Sequences (5'-3')	Tm (°C)
Aspergillus spp.	*18S rRNA*	Fwd.	CCAGCGAGTACATCACCTTGG	59.5
		Rev.	TCCRTTGTTGAAAGTTTTIACTGATT	58.0–60.1
A. fumigatus	*5.8S rRNA*	Fwd.	GGCCCGCCGTTTCGAC	61
		Rev.	GCCCCATACGCTCGAGGA	60.1

Abbreviations: ITS, internal transcribed spacer; I, inosine; R, A or G.

Supplementary Table S2. The specificity of the RF2 mRT-PCR assay for detecting *Pneumocystis jirovecii* and *Aspergillus fumigatus* when challenged using a range of non-respiratory pathogens.

Group	Organism	Source	Catalog No.	*P. jirovecii*	*A. fumigatus*
Bacteria	*Enterococcus faecium*	KCTC	13225	negative	negative
	Enterococcus faecalis	KCTC	5290	negative	negative
	Staphylococcus saprophyticus	KCTC	3345	negative	negative
	Proteus mirabilis	ATCC	29906	negative	negative
	Streptococcus agalactiae	ATCC	13813	negative	negative
	Corynebacterium spp.	ATCC	51860	negative	negative
	Enterobacter aerogenes	ATCC	13048	negative	negative
	Enterobacter cloacae	ATCC	13047	negative	negative
	Lactobacillus jensenii	ATCC	25258	negative	negative
	Lactobacillus gasseri	ATCC	33323	negative	negative
	Lactobacillus crispatus	ATCC	33820	negative	negative
	Klebsiella quasipneumoniae	ATCC	700603	negative	negative
Virus	Coxsackievirus B5	ATCC	VR-185	negative	negative
	Herpes simplex virus	ATCC	VR-734	negative	negative
	BK polyomavirus	ATCC	VR-837	negative	negative
	Parvovirus-B19	NIBSC	12/208	negative	negative

Abbreviations: mRT-PCR, Multiplex real-time PCR; ATCC, American Type Culture Collection; KCTC, Korean Collection for Type Cultures; NIBSC, The National Institute for Biological Standards and Control.

Supplementary Table S3. Distribution of *Aspergillus species* detected using Sanger sequencing

Aspergillus spp.	Number of samples
A. fumigatus	42
A. nidulans	11
A. niger	8
A. flavus	3
A. terreus	1

One sample showed co-infection with *A. fumigatus* and *A. terreus*.
